# Bench Validation of a Compact Low-Flow CO_2_ Removal Device

**DOI:** 10.1186/s40635-018-0200-7

**Published:** 2018-09-24

**Authors:** Alexandra G. May, R. Garrett Jeffries, Brian J. Frankowski, Greg W. Burgreen, William J. Federspiel

**Affiliations:** 1 Department of Chemical and Petroleum Engineering, University of Pittsburgh, Pittsburgh, USA; 2 McGowan Institute for Regenerative Medicine, University of Pittsburgh, 3025 East Carson Street, Suite 226, Pittsburgh, PA 15203 USA; 3 Department of Bioengineering, University of Pittsburgh, Pittsburgh, USA; 4 Computational Fluid Dynamics Group, Center for Advanced Vehicular Systems, Mississippi State University, Mississippi State, MS USA; 5 Department of Critical Care Medicine, University of Pittsburgh Medical Center, Pittsburgh, USA

**Keywords:** Extracorporeal CO_2_ removal, Artificial lung, Acute respiratory distress syndrome, COPD

## Abstract

**Background:**

There is increasing evidence demonstrating the value of partial extracorporeal CO_2_ removal (ECCO_2_R) for the treatment of hypercapnia in patients with acute exacerbations of chronic obstructive pulmonary disease and acute respiratory distress syndrome. Mechanical ventilation has traditionally been used to treat hypercapnia in these patients, however, it has been well-established that aggressive ventilator settings can lead to ventilator-induced lung injury. ECCO_2_R removes CO_2_ independently of the lungs and has been used to permit lung protective ventilation to prevent ventilator-induced lung injury, prevent intubation, and aid in ventilator weaning. The Low-Flow Pittsburgh Ambulatory Lung (LF-PAL) is a low-flow ECCO_2_R device that integrates the fiber bundle (0.65 m^2^) and centrifugal pump into a compact unit to permit patient ambulation.

**Methods:**

A blood analog was used to evaluate the performance of the pump at various impeller rotation rates. In vitro CO_2_ removal tested under normocapnic conditions and 6-h hemolysis testing were completed using bovine blood. Computational fluid dynamics and a mass-transfer model were also used to evaluate the performance of the LF-PAL.

**Results:**

The integrated pump was able to generate flows up to 700 mL/min against the Hemolung 15.5 Fr dual lumen catheter. The maximum vCO_2_ of 105 mL/min was achieved at a blood flow rate of 700 mL/min. The therapeutic index of hemolysis was 0.080 g/(100 min). The normalized index of hemolysis was 0.158 g/(100 L).

**Conclusions:**

The LF-PAL met pumping, CO_2_ removal, and hemolysis design targets and has the potential to enable ambulation while on ECCO_2_R.

## Background

Mechanical ventilation is commonly used to help normalize arterial blood gases in patients with acute hypercapnia but can also contribute to ventilator-induced lung injury (VILI). VILI results from over-distension of the lung, barotrauma, and alveolar damage caused by high volume ventilation [1, 2]. Extracorporeal CO_2_ removal (ECCO_2_R) provides a minimally invasive option to remove CO_2_ independently of the lungs and allow lung rest. ECCO_2_R has been used in patients with acute exacerbations of chronic obstructive pulmonary disease (ae-COPD) to avoid invasive mechanical ventilation, avoid intubation, or assist in extubation and in ventilator weaning [3–5]. In patients with moderate acute respiratory distress syndrome (ARDS), ECCO_2_R has been used in conjunction with lung protective or ultra-protective ventilator settings (tidal volume less than 6 mL/kg [6] or 3 mL/kg [7], respectively) to reduce VILI and correct acidosis [7, 8].

Contemporary ECCO_2_R devices use simplified designs, biocompatible coatings, and polymethylpentene fibers to reduce adverse events [9]. Dual lumen catheters permit single site veno-venous (vv) cannulation and obviate the need for, and risks of, arterio-venous (av) cannulation. A 2016 epidemiological study shows that the trend is toward vv cannulation [10]. The recent focus has been on improving the gas exchange efficiency of ECCO_2_R devices. Active mixing, blood acidification, electrodialysis, and carbonic anhydrase immobilization to the fiber surface are being explored in an effort to reduce fiber surface area and further lower blood flow rates [11–14].

ECCO_2_R utilizes similar principles as extracorporeal membrane oxygenation (ECMO), but with the main goal of removing CO_2_ in patients with otherwise sufficient oxygenation and at a fraction of ECMO blood flow rates. Lower blood flow rates are usable in ECCO_2_R due to the linear slope of the CO_2_ dissociation curve within the physiological pCO_2_ range. Thus, the amount of CO_2_ available per volume of blood decreases linearly with decreasing pCO_2_. Comparatively, the sigmoidal oxy-hemoglobin dissociation curve plateaus at pO_2_ values above 100 mmHg thereby limiting the amount of O_2_ that can be transferred to the blood [9]. Clinically used ECCO_2_R blood flow rates vary from 180 to 1700 mL/min [15] and are classified as either low-flow (< 1 L/min) or mid-flow (1–2 L/min) with ECMO considered high-flow.

Proponents of mid-flow ECCO_2_R contend that higher blood flow rates are required to decrease the likelihood of thrombus formation and to attain the required CO_2_ removal rates. Both of these concerns stem from the velocity of the blood through the device. Research has shown that regions of a device with low-blood velocity are prone to thrombus formations [16], and that increasing the velocity of the blood past the fibers increases the gas exchange efficiency [17]. There are ways, however, to engineer an ECCO_2_R device with increased blood velocity independent of bulk blood flow and permit low-flow ECCO_2_R. The Hemolung RAS and the ultra-low flow ECCO_2_R device (ULFED) each use active mixing technology to increase the blood velocity at the fiber surface while still removing a clinically significant amount of CO_2_ [12, 18]. The Hemolung RAS device has been successfully used at blood flow rates below 500 mL/min to correct hypercapnia in patients [19–21]. The Low-Flow Pittsburgh Ambulatory Lung (LF-PAL) evaluated in this manuscript operates in the low-flow region and uses a narrow bundle cross sectional area to increase blood velocity past the fibers [17].

Here, the performance of the LF-PAL as a low-flow ECCO_2_R device is evaluated through bench studies. The LF-PAL utilizes a 0.65 m^2^ bundle integrated with centrifugal pump into a highly compact device aimed at increasing patient mobility. The CO_2_ removal performance of the LF-PAL was modeled and then measured in vitro at blood flow rates up to 700 mL/min. Additionally, the hydrodynamic performance of the LF-PAL and the resistance of the Hemolung 15.5 Fr catheter were measured and used to determine the anticipated operating conditions. Lastly, in vitro hemolysis was evaluated in the 0.65 m^2^ LF-PAL and compared to two control circuits.

## Methods

### Device description

The LF-PAL incorporates the hollow fiber membrane (HFM) bundle into a highly compact integrated pump-lung. The centrifugal pump drives blood flow from the patient, through the HFM bundle, and back to the patient via a dual lumen catheter located in the jugular vein. The impeller is magnetically coupled to, and driven by, an external motor. The device utilizes a 0.65 m^2^ cylindrical, stacked-type HFM bundle with a diameter of 1.75 in. The bundle is manufactured from polymethypentene fiber sheets (OXYPLUS, Membrana, Wuppertal, Germany) [17]. This prototype device weighs 1850 g and is intended to have the option to be worn by the patient. The specific design and manufacturing details of the LF-PAL devices have been previously published [22]. The device has previously been evaluated for high-flow adult oxygenation [22], but not for low-flow CO_2_ removal.

### CO_2_ removal model

The CO_2_ removal model was based on a previously published mass transfer correlation and assumes radially uniform flow through the bundle [17, 23]. Briefly, the overall CO_2_ mass balance is

1 $$ {Q}_{\mathrm{b}}\frac{{\mathrm{d}C}_{{\mathrm{CO}}_2}}{\mathrm{d}z}=\uppi {R}^2k{a}_{\mathrm{v}}{\Delta P}_{{\mathrm{CO}}_2} $$

where *Q*_b_ is the blood flow rate, ${C}_{{\mathrm{CO}}_2}$ is the total concentration of CO_2_, *z* is the axial coordinate, *R* is the bundle radius, *k* is the mass transfer coefficient, *a*_v_ is the surface area by volume ratio, and ${\Delta P}_{{\mathrm{CO}}_2}$ is the CO_2_ pressure gradient between the sweep gas and blood. The average *P*_CO2_ in the sweep gas was assumed to be 4 mmHg and is based on a previously calculated average of the inlet and outlet sweep gas *P*_CO2_ [23].

A fit of the CO_2_ dissociation curve allows for ${C}_{{\mathrm{CO}}_2}$ to be written as a function of partial pressure [23].

2 $$ {C}_{{\mathrm{CO}}_2}={qP}_{{\mathrm{CO}}_2}^t $$

where *q* and *t* are regression parameters equal to 0.128 and 0.369, respectively. A previously developed [23] mass transfer correlation relating the Sherwood (Sh), Reynolds (Re), and the Schmidt (Sc), numbers was used:

3 $$ \mathrm{Sh}=0.54{\operatorname{Re}}^{0.42}{\mathrm{Sc}}^{1/3} $$

The Sherwood number is defined as $\mathrm{Sh}=\frac{k_{{\mathrm{CO}}_2}{d}_{\mathrm{f}}}{\alpha_{{\mathrm{CO}}_2}{D}_{\mathrm{f}}}$, where *k*_CO2_ is the mass transfer coefficient, *d*_f_ is fiber diameter, ${\upalpha}_{{\mathrm{CO}}_2}$is the solubility of CO_2_ in blood, and *D*_f_ is the facilitated diffusivity. The Reynolds number is defined as $\operatorname{Re}=\frac{\rho\ v}{\varphi\ a\ \mu }$[24], where *ρ* is the fluid density, *v* is the superficial fluid velocity, φ is the cylindrical particle correction factor, *a* is the surface area per volume of the fiber bundle, and *μ* is fluid viscosity. The Schmidt number is defined as $\mathrm{Sc}=\frac{v_{\mathrm{b}}}{D_{\mathrm{eff}}}$ where *v*_b_ is kinematic viscosity, and *D*_eff_ is the effective diffusivity which takes in to account chemically bound CO_2_. The importance of including facilitated diffusion in the calculation and details on the development of these equations have been previously described [23]. An ordinary differential equation solver built into MATLAB (MathWorks, Natick, MA) and based on the Runge-Kutta method was used to solve the differential equation formed by Eqs. 1–3.

Computational fluid dynamics (CFD) was used to analyze the hydraulic and hemodynamic aspects of the LF-PAL device as well as to ensure radially uniform flow through the fiber bundle as assumed by the mass transfer model. Blood flow velocities and pressures within the LF-PAL were modeled via laminar CFD analysis performed using ANSYS Fluent v17 (ANSYS, Canonsburg, PA). Blood was treated as a homogeneous incompressible fluid of density 988 kg/m^3^ and constant viscosity of 3.4 cP. The fiber bundle was modeled as porous media with a uniform viscous resistance [23] of 1e9 m^−2^ and a fluid porosity [25] of 0.58. The CFD mesh consisted of 5.7 M tetrahedral cells, and rotor motion was handled using a frozen relative motion frame of reference.

### In vitro gas exchange

**Fig. 1:**
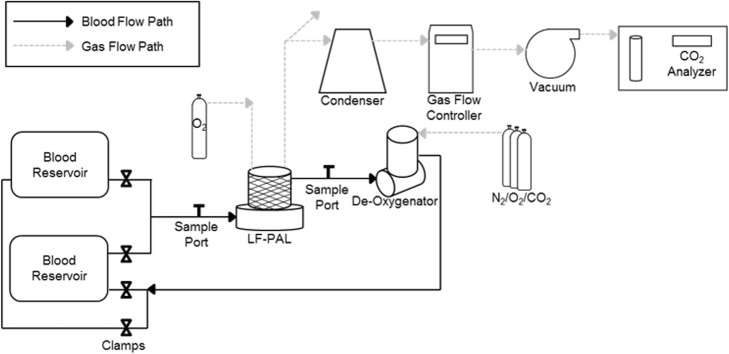
Schematic of the single pass in vitro CO_2_ removal loop. Clamps are used to allow the reservoir filled with venous-conditioned blood to flow through the device and in to the empty second reservoir. Once the first reservoir empties, gases to the de-oxygenator are turned on and the blood is reconditioned to venous conditions

Gas exchange was performed in bovine blood collected from a local slaughterhouse and adhered to the ISO7199 standard [26]. The blood was filtered (40 μm Pall Biomedical, Inc., Fajardo, PR), heparinized (30 U/mL), and treated with gentamicin (0.1 mg/mL). Blood was diluted to a hemoglobin of 12 ± 1 g/dL with phosphate-buffered saline. The test circuit (Fig. 1) consisted of a LF-PAL device, two 6-L compliant blood reservoirs and an Affinity oxygenator (Medtronic, Minneapolis, MN). The reservoir bags were submerged in a water bath to maintain blood temperature at 37 ± 1 °C. The blood was recirculated through a single reservoir while the Affinity oxygenator was used to balance the blood gases to venous conditions. Once the blood was conditioned, clamps were used to divert blood flow through the LF-PAL and into the empty, second reservoir. Blood gas measurements were taken before and after the LF-PAL and analyzed by a Rapidpoint 405 blood gas analyzer (Siemens, Deerfield, IL).

Blood flow rates ranged from 250 to 700 mL/min and were measured by an ultrasonic flow probe (Transonic Systems Inc., Ithaca, NY). Hoffman clamps were used to simulate the resistance of the Hemolung 15.5 Fr dual lumen catheter (ALung Technologies, Pittsburgh, PA). The pressure across the device was monitored with a differential fluid pressure transducer (PX771-025DI; Omega Engineering, Inc., Stamford, CT). The normocapnic condition was tested at an inlet pCO_2_ of 45 ± 5 mmHg and sO_2_ of 65 ± 5%. The gas exchange rate at each flow rate was measured in triplicate.

The oxygen sweep gas flow rate was controlled by a gas flow controller (Fathom Technologies, Georgetown, TX) and ranged from 9 to 19.5 L/min. A WMA-4 CO_2_ analyzer (PP Systems, Amesbury, MA) measured the CO_2_ concentration in the sweep gas (*F*_CO2_) exiting the LF-PAL. Steady state was achieved when the CO_2_ concentration in the sweep gas changed by less than 10 ppm. CO_2_ removal rate (*v*CO_2_) was calculated according to Eq. 4 and normalized to an inlet pCO_2_ of 45 mmHg (*v*CO_2_^*^) according to Eq. 5 [27].

4 $$ {v\mathrm{CO}}_2={\mathrm{Q}}_{\mathrm{SG}}{F}_{\mathrm{CO}2} $$

5 $$ {v\mathrm{CO}}_2^{\ast }={v\mathrm{CO}}_2\frac{45\ \mathrm{mmHg}}{P_{\mathrm{CO}2}^{\mathrm{Inlet}}} $$

where *Q*_SG_ is sweep gas flow rate, *F*_CO2_ is the concentration of CO_2_ in the sweep gas, and ${P}_{\mathrm{CO}2}^{\mathrm{Inlet}}$ is the inlet blood pCO_2_.

### Hydrodynamic performance

The hydrodynamic performance of the 0.65 m^2^ LF-PAL was evaluated using an 8.5 g/L solution of carboxymethylcelluose sodium salt (CMC) (Sigma Aldrich, St. Louis, MO) as the working fluid. The viscosity of the CMC solution at 37 °C was 3.5 cP and verified using a capillary viscometer (Cannon Instrument Company, State College, PA). The LF-PAL was connected to a reservoir submerged in a 37 °C water bath. The rotation rate of the impeller was varied between 800 and 2000 RPM. Hoffman clamps placed at the inlet and outlet to the LF-PAL were used to vary the flow rate between 0 and 1.4 L/min. Pressure was measured at the inlet and outlet to the device using a differential fluid pressure transducer (PX771-025DI; Omega Engineering, Inc., Stamford, CT).

The anticipated catheter for use with the LF-PAL is the Hemolung 15.5 Fr dual lumen catheter. The catheter was inserted into a 1600-mL reservoir bag, and pressure was measured at the inflow and outflow ports of the catheter using a differential fluid pressure transducer. Pressure within the reservoir was assumed to be spatially uniform so that the resistance of the catheter may be calculated as the pressure difference between the inflow and outflow tubing connection ports of the catheter. Flow was driven by a Biomedicus BP-80 pump (Medtronic, Minneapolis, MN) and ranged between 100 and 900 mL/min.

### In vitro hemolysis

Bovine blood was collected and prepared as in the gas exchange experiments. The hemolysis test circuit consisted of the LF-PAL and the Hemolung 15.5 Fr dual lumen femoral catheter in order to reflect clinical use. The LF-PAL was tested at 500 mL/min.

The control circuit replaced the LF-PAL with a PediMag pump (Thoratec, Pleasanton, CA) and Minimax PLUS Hollow Fiber Oxygenator (Medtronic, Minneapolis, MN) and was run in parallel with the test circuit. The control circuit was operated at 1500 mL/min (3750–3800 RPM) to match the CO_2_ removal rates of the LF-PAL operated at 500 mL/min [28]. Hoffman clamps were used to simulate inclusion of a 12 Fr arterial cannula (Medtronic Bio-Medicus Cannula #96820-012) and 14 Fr venous cannula (Medtronic Bio-Medicus Cannula #96830-014) [29, 30]. Both circuits contained an 800-mL compliant blood reservoir (Medtronic; Minneapolis, MN) submerged in a water bath to maintain blood temperature at 37 ± 2 °C.

Blood samples from each circuit were taken every 30 min over a 6-h period to measure plasma free hemoglobin (PfHb), hematocrit, and hemoglobin. Details of the sampling and PfHb measurement methods, calculation of the normalized index of hemolysis (NIH), and therapeutic index of hemolysis (TIH) have been previously published [12, 17]. Three independent trials were conducted for each circuit. The results of a second control (Medtronic Biomedicus BP-50, Minimax, and Medtronic Bio-Medicus Cannulas) are also included and methods have been previously described by our group [12]. This second control, BP-50 control, was operated at the blood flow rate required for the Minimax to meet our 70 mL/min CO_2_ removal target.

### Statistics

Calculations for statistical comparisons were completed using SPSS (IBM, Armonk, NY). A one-way ANOVA with Tukey HSD post hoc analysis was used to compare the mean TIH values and the mean NIH values. Levene’s test was used to test for homogeneity of variances. The effect of device type was considered significant. Comparisons between the three TIH and NIH of the devices were considered significant at *p* < 0.05.

## Results

### Model and in vitro CO_2_ removal

**Fig. 2:**
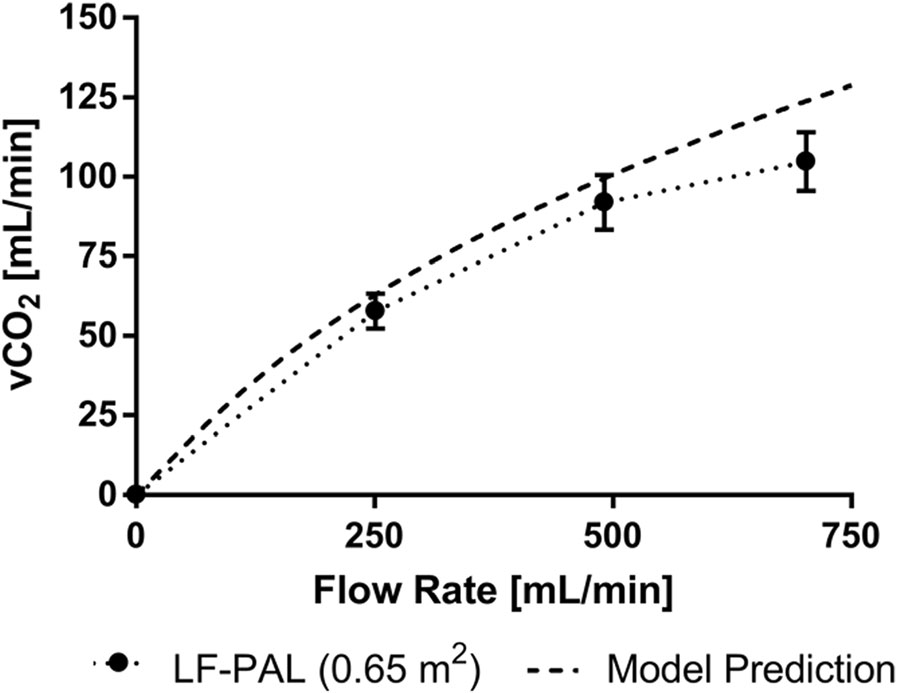
In vitro CO_2_ removal data of the LF-PAL. The model predicted CO_2_ removal rate is also plotted. The maximum CO_2_ removal rate for the LF-PAL was 105 mL/min. The model predicted the performance between 7.7–15.4% of the in vitro results

In vitro gas transfer results and model predictions of the 0.65-m^2^ bundle are shown in Fig. 2. The CO_2_ removal rate increased with increasing blood flow rate. The maximum CO_2_ removal rate of the LF-PAL was 105 ± 9.2 mL/min at a blood flow rate of 703 mL/min. The model predicted CO_2_ removal rates were within 7.7–15.4% of the experimental values.

### Pump requirements

**Fig. 3:**
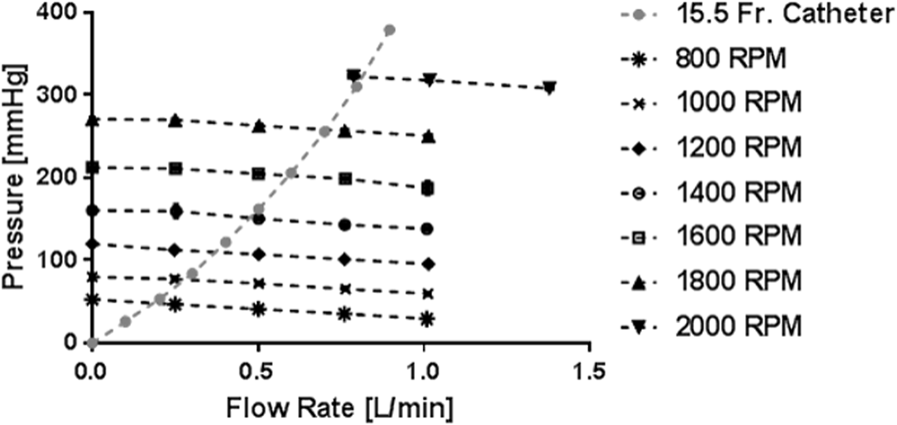
The pressure generated by the LF-PAL at impeller rotation rates of 800, 1000, 1200, 1400, 1600, 1800, and 2000 RPM between 0 and 1 L/min. The pressure requirements of the 15.5 Fr dual lumen catheter are also shown

The pressure generated by the 0.65 m^2^ LF-PAL device is shown in Fig. 3. The pressure requirements for operation with the 15.5 Fr dual lumen catheter are also shown in Fig. 3. The 0.65 m^2^ LF-PAL reached the required flow rate range of 250 to 700 mL/min at impeller rotation rates between 800 and 1800 RPM.

**Fig. 4:**
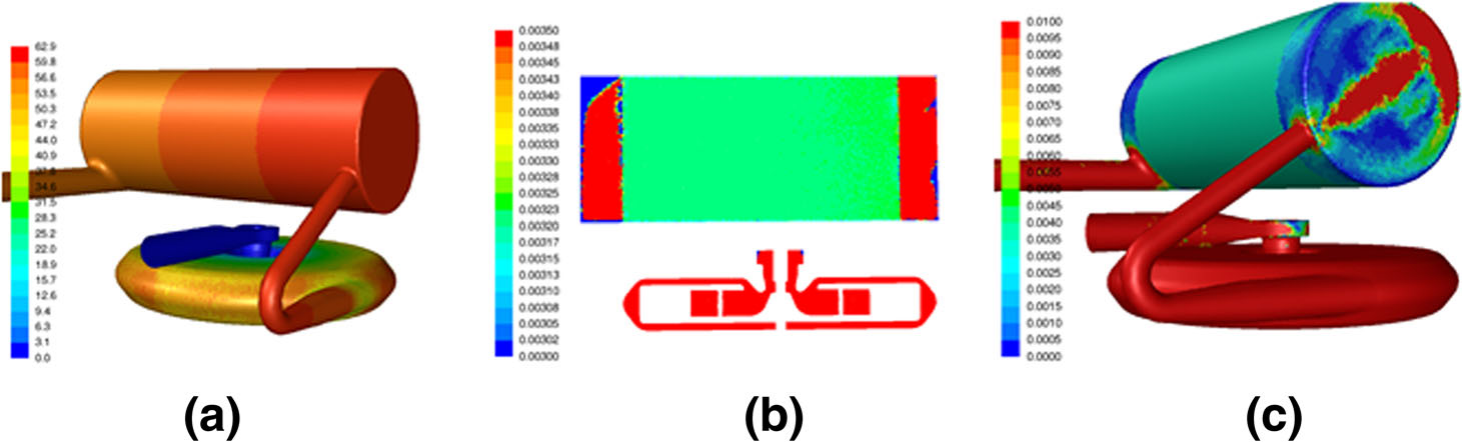
CFD analysis results for 850 RPM and 0.25 L/min showing (**a**) predicted pressure (mmHg) throughout the device, (**b**) fluid velocity (m/s) through the fiber bundle, and (**c**) near wall velocity magnitudes (m/s) on the device surfaces

Typical CFD results shown in Fig. 4 for the 0.65 m^2^ LF-PAL demonstrate adequate pressure generation and uniform flow distribution throughout the bundle. Generated pressure heads predicted by CFD were 51 mmHg for 850 RPM, 0.25 L/min and 265 mmHg for 1870 RPM, 0.70 L/min. The maximum shear stress predicted by CFD in the device for these operating conditions was located on the rotor surfaces and was less than 200 Pa. Flow through the fiber bundle was shown to have a very uniform distribution and exhibited no flow separation. For both operating conditions, the LF-PAL was shown to have the slowest flow occurring in the inflow elbow and the bundle inlet and outlet plenums.

### In vitro hemolysis

Table 1 provides the TIH and NIH values for the 0.65 m^2^ LF-PAL and control circuits. The rate of PfHb increase over time was linear (R^2^ > 0.90) for the LF-PAL and control circuit. The TIH of the LF-PAL (0.08 ± 0.017 g/100 min), the Pedimag control (0.043 ± 0.0004 g/100 min), and the BP-50 control (0.123 ± 0.013 g/100 min) all significantly differed from one another (*p* < 0.05). The NIH of the LF-PAL (0.158 ± 0.034 g/100 L), the Pedimag control (0.029 ± 0.003 g/100 L), and the BP-50 control (0.105 ± 0.012 g/100 L) all significantly differed from one another (*p* < 0.05).

**Table 1:** In vitro hemolysis of the LF-PAL

Device	Flow Rate	NIH	TIH
	[mL/min]	[g/100 L]	[g/100 min]
LF-PAL (0.65 m^2^)	500	0.158 ± 0.034^†^	0.080 ± 0.017^‡^
Pedimag control	1500	0.029 ± 0.003^†^	0.043 ± 0.004^‡^
BP-50 control	1250	0.105 ± 0.012^†^	0.123 ± 0.013^‡^

^†^Statistically significant (*p* < 0.05) compared to other devices

^‡^Statistically significant (*p* < 0.05) compared to other devices

## Discussion

Clinical evidence demonstrates that ECCO_2_R can prevent the need for intubation, allow for protective and ultra-protective lung ventilation, and aid in weaning patients from mechanical ventilation [6, 31]. Low-flow ECCO_2_R devices aim to provide minimally invasive, complementary treatment options for ae-COPD patients and patients with moderate ARDS requiring mechanical ventilation. This manuscript details the in vitro and computational characterization of the LF-PAL for ECCO_2_R. The LF-PAL is an integrated pump-lung designed to allow simplified patient ambulation while on ECCO_2_R. The LF-PAL removed up to 105 mL/min of CO_2_ thereby exceeding the 70 mL/min target and had acceptable hemolysis.

ECCO_2_R has been applied to a variety of clinical applications including weaning patients from mechanical ventilation, avoiding intubation, and permitting lung protective ventilation [3–5, 7, 8]. As a result of the range of clinical applications, the CO_2_ removal rate required for ECCO_2_R is not well defined. Additionally, CO_2_ removal is dependent on the total CO_2_ content of the blood. Hence, the degree of hypercapnia will affect the CO_2_ removal rate at a given blood flow rate. The CO_2_ removal rate, however, will proportionally increase with increases in pCO_2_. The CO_2_ removal target must therefore be reported as a percentage of the rate of metabolically produced CO_2_ or the pCO_2_ of the blood entering the device must be specified. Trahanas et al. provide a review of studies since 2009 of ECCO_2_R used in hypercapnic COPD patients and CO_2_ removal rates ranged from 80 to 160 mL/min [32]. From this, the authors proposed that an ambulatory ECCO_2_R device must remove at least half of the metabolic CO_2_. Under normocapnic conditions, this would be approximately 100 mL/min. Commercial ECCO_2_R devices report removal rates of 20–40% of the metabolically produced CO_2_ [33–35]. Under normocapnic conditions, these rates are equal to 40–80 mL/min. Based on this data, we set 70 mL/min as the minimum target CO_2_ removal rate for the LF-PAL at normocapnia. The LF-PAL exceeded this target at low-flow ECCO_2_R blood flow rates. Recent, on-going, and upcoming ECCO_2_R clinical trials (XTRAVENT [7], REST (NCT02654327), SUPERNOVA (NCT02282657), and VENT-AVOID (NCT03255057)) should provide a more defined CO_2_ removal target for devices in development.

Concerns with low-flow ECCO_2_R, compared to mid-flow, include inadequate CO_2_ removal and thrombus formation resulting from low-velocity regions within the device [16]. To mitigate both of these effects, the LF-PAL uses a fiber bundle with a narrow cross sectional area to increase local blood velocities and achieve clinically significant CO_2_ removal. In addition, CFD results of the LF-PAL demonstrate uniform blood velocity through the bundle. Other low-flow devices use active mixing technology to increase local blood velocity in an effort to achieve required CO_2_ removal rates. The Hemolung incorporates a rotating core [18], and the ULFED uses rotating impellers [12]. The drawback is that too high of an increase in blood velocity may detrimentally increase hemolysis. The Hemolung, however, has been used in humans without causing clinically significant hemolysis, though no in vitro hemolysis data are available for comparison [20].

At least one center has begun ambulating ECCO_2_R patients to reduce muscle deconditioning and allow for greater physical therapy [3]. A compact device which does not require a saline infusion or vacuum pump, such as the LF-PAL, that could also be worn would simplify ambulation. Current, clinically used ECCO_2_R devices are portable, but are not designed to be worn by the patient [36–40]. The jugular cannulation and cartridge design of the Hemolung RAS permits ambulation. The device, however, must reside on the roller cart, which houses the required saline infusion and gas side vacuum pump and does not provide the option to be worn by the patient. The arterio-venous CO_2_ removal (AVCO2R) device is also under development as a wearable ECCO_2_R device [41]. The av-cannulation of the AVCO2R, however, relies on the patient’s cardiovascular system to drive blood flow. The CO_2_ removal rate is therefore dependent on the cardiac function of the patient [42]. The vv-cannulation and pump-driven blood flow of the LF-PAL allows the clinician greater control of the extracorporeal blood flow and, in turn, the CO_2_ removal rate. The compact design and dual lumen cannulation of the LF-PAL lends itself to ambulation.

In this study the hemolysis of the LF-PAL was only evaluated at 500 mL/min. Shear stress within the circuit will increase as blood flow increases and likely result in elevated hemolysis at higher blood flow rates. Thus, when the LF-PAL is operated at the maximum blood flow rate, 700 mL/min, the TIH of the LF-PAL will likely increase, as would the TIH of the control circuit when operated at a higher blood flow rate. A limitation to the TIH is the lack of an established threshold value correlated to clinically significant hemolysis in vivo. Thus, in vitro studies are limited to a comparative assessment between two circuits. Future in vivo studies will thoroughly evaluate if the hemolysis generated by the LF-PAL is clinically significant in addition to any effect the device may have on platelet activation or end organ function.

## Conclusion

Evidence demonstrating the benefits of partial CO_2_ removal by ECCO_2_R systems in conjunction with non-invasive ventilation or lung protective ventilation continues to grow. The LF-PAL provides the CO_2_ removal benefits of low-flow ECCO_2_R in a compact design. Future work will focus on 7-day in vivo studies to further characterize the LF-PAL performance and the effect of the device on the cardiopulmonary system.

## Abbreviations

ae-COPD: Acute exacerbation of chronic obstructive pulmonary disease
ARDS: Acute respiratory distress syndrome
av: Arterio-venous
CFD: Computational fluid dynamics
ECCO_2_R: Extracorporeal CO_2_ removal
ECMO: Extracorporeal membrane oxygenation
LF-PAL: Low-Flow Pittsburgh Ambulatory Lung
NIH: Normalized index of hemolysis
pfHb: Plasma free hemoglobin
TIH: Therapeutic index of hemolysis
ULFED: Ultra-low flow extracorporeal device
VILI: Ventilator-induced lung injury
vv: Veno-venous
